# A Case Report of Clozapine-Induced Cardiomyopathy

**DOI:** 10.7759/cureus.87032

**Published:** 2025-06-30

**Authors:** Gourav Gourisaria, Navin Sumanasan, Goksu Ozen, Thejas Swaroop Konduru, Pradeep Karunakaran Thozhuthumparambil

**Affiliations:** 1 Acute Internal Medicine, Sandwell and West Birmingham Hospitals NHS Trust, Birmingham, GBR; 2 Endocrinology and Diabetes, Royal Glamorgan Hospital, Cardiff, GBR

**Keywords:** antipsychotic side effects, cardiomyopathy, clozapine, clozapine monitoring, drug-induced heart failure, myocarditis, schizoaffective disorder

## Abstract

Clozapine is an antipsychotic medication prescribed for patients who do not respond to or cannot tolerate standard antipsychotic treatments. It is also used to manage psychosis associated with Parkinson’s disease. As a high-risk drug, clozapine requires strict monitoring. A rare but serious side effect is cardiomyopathy, which necessitates discontinuation of the medication. This report presents the case of a 42-year-old male patient admitted with recurrent falls and urinary sepsis while on clozapine. He exhibited persistent tachycardia and drowsiness and was diagnosed with cardiomyopathy, likely induced by clozapine.

## Introduction

Clozapine is primarily indicated for treatment-resistant schizophrenia and related psychiatric disorders [1]. It is also effective in managing psychosis associated with Parkinson’s disease [2]. Although long-term clozapine therapy is linked to reduced all-cause mortality and lower suicide rates, it carries the risk of severe adverse effects, including myocarditis, cardiomyopathy, orthostatic hypotension, and tachycardia [3,4]. The reported incidence rates of cardiomyopathy differ widely, ranging from 0.06% in a French study [5] to 3.88% in a contemporary Australian cohort study [6]. These discrepancies may reflect challenges such as the nonspecific clinical features of cardiomyopathy, lack of diagnostic standardization, and variability in adverse event reporting systems [7]. In cases where clozapine-induced cardiomyopathy is suspected, early echocardiographic evaluation and cardiology referral are recommended [8]. This case report describes a 42-year-old male who developed cardiomyopathy attributed to clozapine, highlighting the importance of timely recognition and management.

## Case presentation

A 42-year-old male with schizoaffective disorder and type 2 diabetes presented to the emergency department with palpitations, recurrent collapses, and shortness of breath. He denied other cardiac or abdominal symptoms and reported no recent travel history. He had no prior cardiac history; however, there was a strong family history of myocardial infarction in his mother and father. His medications included clozapine (125 mg in the morning and 225 mg in the evening), sodium valproate (250 mg in the morning and 1,500 mg in the evening), metformin (500 mg twice daily), and atorvastatin (40 mg daily).

On examination, the patient appeared unwell and disoriented. Chest auscultation revealed clear lungs with normal heart sounds. Abdominal examination was unremarkable. Laboratory tests revealed leucocytosis with raised CRP, as shown in Table 1. Vital signs showed a heart rate of 90-110 bpm, oxygen saturation of 90% on room air, a normal respiratory rate, and a mean arterial pressure of approximately 100 mmHg. Urinalysis indicated infection, and intravenous gentamicin was initiated. Electrocardiogram (ECG) showed sinus tachycardia with T-wave inversion in the anterior leads with normal QTc. Troponin and brain natriuretic peptide (BNP) levels were within normal limits. Arterial blood gas analysis revealed type 2 respiratory failure. A computed tomography (CT) pulmonary angiogram ruled out pulmonary embolism. Bedside ultrasound detected abdominal free fluid, and contrast-enhanced CT of the abdomen revealed no acute pathology. Urine cultures confirmed *Escherichia coli *infection.

**Table 1:** Summary of admission investigations CRP: C-reactive protein, MCS: microscopy, culture, and sensitivity, *E.coli*: *Escherichia coli*, RSV: respiratory syncytial virus, SARS‑CoV‑2: severe acute respiratory syndrome coronavirus 2, NT-proBNP: N-terminal pro B-type natriuretic peptide, INR: international normalised ratio, eGFR: estimated glomerular filtration rate, ALT: alanine aminotransferase, PCR: polymerase chain reaction

Laboratory tests		Value	Reference range
Full blood count	White blood cell (x 10^9^/L)	15.2	(4.00-11.00)
	Red blood cell (x 10^12^/L)	4.92	(4.5-6.00)
	Haemoglobin (g/L)	140	(125-180)
	Haematocrit (L/L)	0.43	(0.4-0.5)
	Neutrophils (x 10^9^/L)	12.92	(1.70-7.50)
	Lymphocytes (x 10^9^/L)	1.16	(1.00-4.00)
	Monocytes (x 10^9^/L)	1.02	(0.2-0.8)
	Basophil (x 10^9^/L)	0.05	(0.00-0.10)
	Eosinophil (x 10^9^/L)	0.06	(0.1-0.4)
	Platelet (x 10^9^/L)	149	(150-400)
Coagulation screen	INR	1.1	(0.80-1.20)
Urea and electrolytes	Sodium (mmol/L)	136	(135-145)
	Potassium (mmol/L)	4.2	(3.5-5.5)
	Urea (mmol/L)	4.7	(2.5-7.8)
	eGFR (mL/min)	>90	(>90)
	Creatinine (umol/L)	65	(59-104)
Cardiac enzyme	Troponin I (ng/L)	5.7	(0-4.9)
	NT-proBNP (ng/L)	93	(<400)
Inflammatory marker	CRP (mg/L)	219	(<5)
Liver function tests	Bilirubin (umol/L)	13	(<21)
	ALT (U/L)	29	(<45)
Haematinics	Ferritin (ug/L)	157	(22-275)
	Vitamin B12 (ng/L)	>128	(51-128)
	Folate (ug/L)	4.2	(3.1-20.0)
Drug levels	Clozapine level (mg/L)	689	(350-500)
	Valproate level (mg/L)	44.8	(50-100)
Miscellaneous	Blood culture	Both aerobic and anaerobic bottles showed no growth	
	Urine MCS	E.coli grown	
	SARS CoV-2 PCR	Negative	
	Influenza A and B and RSV PCR	Negative	

**Figure 1:**
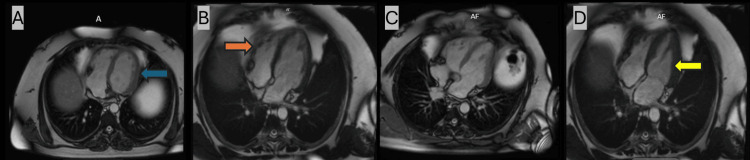
Coronal images of cardiovascular magnetic resonance (CMR) (A) The left ventricle (LV) wall thickness is normal (denoted by the blue arrow). (B) The right ventricle (RV) is normal in size (denoted by the orange arrow). (C and D) The LV is dilated in systole (denoted by the yellow arrow).

The patient's condition worsened, with drowsiness, persistent tachycardia, and hypoxia requiring oxygen. A head CT showed no abnormalities. It was discovered that the patient had been taking twice his prescribed dose of sodium valproate (3.5 g/day). His valproate level was low, the reason for which was unclear, but his clozapine level was elevated. Given the elevated clozapine level and symptoms, clozapine-induced cardiomyopathy was suspected, and the dose was reduced. Clozapine-induced myocarditis was a part of the differentials but was less likely given the normal cardiac biomarkers. Echocardiography showed a reduced ejection fraction (36-40%) with a normal right ventricle and valves. Clozapine was discontinued after psychiatric review, and olanzapine (5 mg nightly) was initiated. Cardiovascular magnetic resonance (CMR) imaging confirmed hypokinetic non-dilated cardiomyopathy (HNDC) with moderate left ventricular systolic dysfunction and an ejection fraction of 40%, with no infarction or scarring, as shown in Figures 1A-1D. The patient improved, was weaned off oxygen, and regained full consciousness. The cause of his presentation was clozapine-induced cardiomyopathy, and he was treated with dapagliflozin and losartan. He was discharged on olanzapine and sodium valproate, with ongoing follow-up by cardiology and mental health services.

The cardiology team decided to proceed with cardiac catheterization, and the patient underwent an elective diagnostic invasive coronary angiogram to rule out any fixed coronary artery disease. This showed non-obstructed coronaries. In the absence of any significant coronary plaques or structural heart disease, the diagnosis of cardiomyopathy secondary to clozapine was made. The patient was referred to cardiac rehabilitation, and he was clinically improving with the medications and rehab. He has not had a repeat estimation of his cardiac function as he continues to be well in himself and asymptomatic.

## Discussion

Clozapine is often highly effective in treating schizophrenia that is resistant to other antipsychotic medications and is known to significantly reduce suicidal behavior [1-4,7,8]. However, its use is associated with serious adverse effects, including myocarditis and cardiomyopathy, both of which are challenging to diagnose early and can lead to severe clinical outcomes [3]. The reported incidence of clozapine-induced cardiomyopathy ranges from 0.02% to 0.1%, with mortality rates as high as 17.9% [9]. While routine monitoring for agranulocytosis and metabolic syndrome is well established, cardiac surveillance is equally important. Regular electrocardiograms (ECGs) are recommended due to clozapine’s known risk of QTc interval prolongation [10].

Valproic acid has previously been identified as a potential risk factor for clozapine-induced myocarditis, and a French cohort study found that 50% of affected patients were concurrently taking valproate [5]. In this case, the patient was receiving a higher-than-recommended dose of valproic acid, which further supported the diagnosis of clozapine-induced cardiomyopathy.

Cardiomyopathy related to clozapine typically manifests early in the treatment course, often within the first few weeks [11]. Although the exact mechanism is unclear, several hypotheses have been proposed. Some studies implicate dysfunction in the CYP1A2 and CYP3A4 enzyme systems, which play a role in clozapine metabolism [12]. Other evidence suggests a link between selenium deficiency and increased susceptibility to cardiomyopathy [13]. During the initial four weeks of therapy, patients should be monitored closely for signs such as fatigue, chest discomfort, and dyspnea, along with vital signs and relevant laboratory markers. Elevated C-reactive protein levels (>100 mg/L) and troponin values ≥2 times the upper normal limit provide 100% sensitivity for identifying myocarditis in symptomatic individuals [14].

Published data indicate that myocarditis often resolves both symptomatically and biochemically following clozapine discontinuation [15]. Furthermore, cessation of clozapine therapy when left ventricular ejection fraction (LVEF) remains above 40% is associated with a favorable prognosis and potential for full recovery [16]. In contrast, patients with significantly reduced LVEF have a poorer outlook. This case highlights the importance of obtaining a thorough medication history, including assessment of dosing accuracy and adherence. A high index of suspicion, coupled with timely cardiac assessment, was critical to the diagnosis and effective management of clozapine-induced cardiomyopathy in this patient.

## Conclusions

Clozapine remains a cornerstone treatment for refractory schizophrenia, but it carries the risk of rare yet serious cardiac complications, including myocarditis and cardiomyopathy. Early identification of these adverse effects and immediate cessation of the drug are critical to improving outcomes. Initial investigations, such as C-reactive protein, troponin, and serum drug levels, can assist in timely diagnosis. This case underscores the necessity for ongoing cardiac monitoring in patients receiving clozapine and emphasizes the value of close collaboration between psychiatry and cardiology to ensure safe and effective management.
